# Anti-Angiogenic Effects of a Mutant Endostatin: A New Prospect for Treating Retinal and Choroidal Neovascularization

**DOI:** 10.1371/journal.pone.0112448

**Published:** 2014-11-07

**Authors:** Yujing Bai, Min Zhao, Chunfang Zhang, Shanshan Li, Yun Qi, Bin Wang, Lvzhen Huang, Xiaoxin Li

**Affiliations:** 1 Key Laboratory of Vision Loss and Restoration, Ministry of Education, Beijing Key Laboratory for the Diagnosis and Treatment of Retinal and Choroid Diseases, Department of Ophthalmology, Peking University People's Hospital, Beijing, People's Republic of China; 2 Clinical Epidemiology & Biostatistics, Peking University People's Hospital, Beijing, People's Republic of China; Ottawa Hospital Research Institute, Canada

## Abstract

**Purpose:**

Pathological fundus angiogenesis is a major cause of vision loss in retina diseases. Endostatin, a C-terminal fragment of collagen XVIII, is an endogenous anti-angiogenic protein. The present study aimed to investigate the *in vitro* and *in vivo* anti-angiogenic properties of two proteins: an N-terminal H1D/H3D mutant endostatin (M-ES) and a polyethylene glycol propionaldehyde (PEG) covalent M-ES (PEG-M-ES).

**Methods:**

M-ES and PEG-M-ES properties were characterized *in vitro* using a zinc ion binding assay and a stability test. Activity assays, including migration, proliferation, and tube formation assays, were performed with human retinal microvascular endothelial cells (HRMECs) and human umbilical vein endothelial cells (HUVECs). Mouse oxygen-induced retinopathy (OIR) and choroidal neovascularization (CNV) models were used to evaluate *in vivo* anti-angiogenic effects. In addition, a rabbit model was used to study the retinal pharmacokinetic profile following an intravitreal injection.

**Results:**

The results indicated that the H1D/H3D mutations of endostatin reduced the zinc binding capacity of M-ES and facilitated PEG covalent binding. PEG-M-ES was more stable and persisted longer in the retina compared with M-ES. The *in vitro* studies demonstrated that M-ES and PEG-M-ES inhibited HRMEC and HUVEC proliferation, migration, and tube formation more efficiently than ES. *In vivo*, a single intravitreal injection of M-ES and PEG-M-ES significantly decreased neovascularization in both the OIR and CNV animal models.

**Conclusion:**

The present study demonstrated for the first time that PEG-M-ES exhibits a long-term inhibitory effect on neovascularization *in vitro* and *in vivo*. These data suggest that PEG-M-ES may represent an innovative therapeutic strategy to prevent fundus neovascularization.

## Introduction

Retinal and choroidal neovascularization are common symptoms in ocular diseases, and their incidence rates have recently increased [1]. These diseases include retinopathy of prematurity (ROP), high myopia macular degeneration, and age-related macular degeneration (AMD), which mainly affect infants, young adults, and the elderly, respectively [2]–[4]. Complications resulting from uncontrolled fundus neovascularization are the major causes of severe and irreversible vision loss worldwide [5], [6].

Endostatin is an endogenous 20-kDa C-terminal fragment of collagen XVIII that exhibits potent angiogenic inhibitory effects [7]. Since the anti-angiogenic effects of endostatin were first reported by Folkman and colleagues in 1997 [8], researchers worldwide have investigated endostatin as a potential target for anti-tumor therapy. Recently, it has been demonstrated in clinical and laboratory settings that endostatin inhibits the progression of more than 60 types of tumors [9]. Furthermore, research efforts have demonstrated that endostatin's antitumor effects result from a broad spectrum of mechanisms, including binding to cell surface receptors, triggering intracellular signaling networks, and regulating the secretion of angiogenic and anti-angiogenic genes [10].

Polyethylene glycol (PEG) is a polyether compound with broad applications in medicine [11]. PEG can serve as a protein carrier given its long half-life, stable tissue concentrations, enhanced biocompatibility, and low biodegradability [12]. As early as the 1990s, PEG-modified adenosine deaminase (PEG-ADA) was commercially available for pharmaceutical use [13], resulting in the subsequent production of PEGylated interferon alfa-2a and alfa-2b as well as the PEGylated anti-vascular endothelial growth factor (VEGF) medication pegaptanib [14], [15]. The promising effects of PEG provided researchers with useful methods for protein modification.

Previously, we demonstrated that native endostatin inhibits retinal angiogenesis [16]. To explore more potent anti-angiogenic proteins, we synthesized an N-terminal H1D/H3D mutant endostatin (M-ES) in the present study. Then, we covalently attached PEG to the H1D/H3D M-ES (PEG-M-ES). To investigate the anti-angiogenic properties of M-ES and PEG-M-ES *in vitro* and *in vivo*, we performed a series of assays to characterize the proteins, including a zinc ion binding assay and a stability test. We also conducted a series of activity assays (i.e., migration, proliferation, and tube formation assays). Mouse oxygen-induced retinopathy (OIR) and choroidal neovascularization (CNV) models were used to evaluate *in vivo* anti-angiogenic effects. In addition, a rabbit model was used to study the retinal pharmacokinetic profile following an intravitreal injection. Our encouraging results demonstrate for the first time that PEG-M-ES has a long-term angiogenic inhibitory effect on fundus neovascularization both *in vitro* and *in vivo* and may represent an innovative therapeutic strategy.

## Materials and Methods

### Cell lines and animals

Human retinal microvascular endothelial cells (HRMECs) (Angio-Proteomie, Boston, MA, US) and human umbilical vein endothelial cells (HUVECs) (American Tissue Culture Collection, Manassas, VA, US) [17] were used for the *in vitro* study. HRMECs were cultured in special endothelial growth medium (Endothelial Growth Medium, Cat# cAP-02, Angio-Proteomie, Boston, MA, US) and HUVECs were cultured in Dulbecco's modified Eagle medium (Nutrient Mixture F-12, DMEM/F-12, Gibco, Grand Island, NY, US) with 10% fetal bovine serum (FBS; Gibco, Grand Island, NY, US) as recommended by the manufacturer. Neonatal mice (C57BL/6J) and adult mice (C57BL/6J, 20∼25 g) were purchased from Vital River Laboratory Animal Technology Company (Beijing, China). Adult New Zealand rabbits (female, 2-2.5 kg) were purchased from the Chinese People's Liberation Army Military Academy of Medical Sciences (Beijing, China). The animals were raised in the Peking University People's Hospital. To induce deep anesthesia, a cocktail of ketamine and xylazine (50 mg/kg ketamine and 10 mg/kg xylazine) was used. This study adhered to the ARVO Statement for the Use of Animals in Ophthalmic and Vision Research and was performed in accordance with the guidelines provided by the Animal Care Use Committee (IACUC) of Peking University. The IACUC and the ethics committee of Peking University People's Hospital approved this study.

### Cloning, expression, and purification of human mutant endostatin (M-ES)

The M-ES encoding gene (Table 1) was sub-cloned into the expression vector pET-30a (+) plasmid between the NdeI and EcoRI restriction sites (Kit Lot No. N72770, Novagen, Germany). The recombinant plasmids were introduced into *E. coli* DH5α competent cells (Code No. D9057, Takara Bio Inc., Japan) to obtain recombinant bacteria. Positive clones were selected and inoculated into Luria-Bertani (LB) broth. Isopropyl-D-thiogalactopyranoside (IPTG) was added to the broth and incubated for another 4 h at 37°C while shaking at 230 rpm. *E. coli* cells were harvested by centrifugation, and 150 mM NaCl Tris-HCL buffer (pH 9.0, 50 mM) was added to obtain the cell suspension. Then, sonication was performed. M-ES was expressed in the inclusion body protein products and was subjected to purification, renaturation, and repurification procedures. Purified M-ES proteins were detected by sodium dodecyl sulfate polyacrylamide gel electrophoresis (SDS-PAGE).

**Table 1:** M-ES nucleotide sequence.

atggacagcgaccgtgatttccagccggtgctccacctggttgcgctcaacagccccctgtcaggcggcatgcggggcatccgcggggccgacttccagtgcttccagcaggcgcgggccgtggggctggcgggcaccttccgcgccttcctgtcctcgcgcctgcaggacctgtacagcatcgtgcgccgtgccgaccgcgcagccgtgcccatcgtcaacctcaaggacgagctgctgtttcccagctgggaggctctgttctcaggctctgagggtccgctgaagcccggggcacgcatcttctcctttgacggcaaggacgtcctgaggcaccccacctggccccagaagagcgtgtggcatggctcggaccccaacgggcgcaggctgaccgagagctactgtgagacgtggcggacggaggctccctcggccacgggccaggcctcctcgctgctggggggcaggctcctggggcagagtgccgcgagctgccatcacgcctacatcgtgctctgcattgagaacagcttcatgactgcctccaagtag

### PEGylated endostatin mutant preparation

Monomethoxy polyethylene glycol propionaldehyde (PEG, molecular weight 20 kDa, Sigma, US) was added to the recombinant human M-ES solution, and the reducing agent NaHBCN was added at a final concentration of 20 mM. The reaction solution was stirred at room temperature for 4 h, and a cation exchange column was used to separate and purify the PEG-M-ES. Based on the charge strength of the modified and unmodified protein, M-ES and PEG-M-ES were eluted. The collected proteins were detected by SDS-PAGE. PEGylated wild type endostatin (PEG-W-ES) was prepared using the same procedure described above for PEG-M-ES.

### Analysis of zinc ion concentration based on the zinc ion binding assay

Zinc ions are important for the stability of protein. To determine the concentration of zinc ions, an atomic absorption spectrophotometer (Hitachi Z 8230 Polarized Zeeman) was used. Wild type endostatin (W-ES) and M-ES were diluted to 1 µM with Tris-HCl (pH 7.4, 5 mM). Ethylenediaminetetraacetic acid (EDTA) and ZnCl_2_ were added to the W-ES and M-ES test solutions to a final concentration of 100 µM. After incubation for 10 h, Tris-HCl buffer (pH 7.4, 5 mM) was added to dialyze the test solution; then, the zinc ion concentration was detected. The experiments were repeated five times.

### PEG stability test

PEG-M-ES and PEG-W-ES were diluted with 10 mM Tris-HCl (pH 8.0) to 1 mg/ml and added to sterile vials. After performing a 37°C acceleration test for 7 and 15 d, SDS-PAGE was used to detect the shedding of PEG from PEG-M-ES and PEG-W-ES. The degradation rate was calculated using the following equation: Degradation rate (%)  =  degradation bands signal/(degradation bands signal + target bands signal) ×100. The experiments were repeated five times.

### Endothelial cell proliferation assays

The effects of ES, M-ES, and PEG-M-ES on cell proliferation were studied using a BrdU Cell Proliferation Assay Kit (#6813, Cell Signaling Technology) as previously described [18]. Briefly, HRMECs and HUVECs were seeded at a density of 1×10^4^ cells per well in 96-well plates. Then, 100 µl of ES, M-ES, or PEG-M-ES (10^−4^ mg/ml or 10^−6^ mg/ml without FBS containing culture medium) with 20 ng/ml of VEGF was incubated for an additional 48 or 72 h. At the indicated time points, the proliferation assays were performed according to the manufacturer's instructions. Each experiment was performed in five wells and repeated at least three times.

### Endothelial cell Transwell migration assay

A Transwell migration assay (Corning, US, Cat# 3422) was used as previously described to evaluate the migration capacity of endothelial cells [16]. Briefly, 2×10^4^ cells were placed in the top chamber in 200 µl of serum-free medium. Culture medium with 10^−4^ mg/ml and 10^−6^ mg/ml of ES, M-ES or PEG-M-ES and 20 ng/ml of VEGF were placed in the bottom chamber at a final volume of 600 µl. All migration assays were conducted at 37°C for 4 h. At the end of the assay, the cells were fixed in 4% PFA and stained with DAPI (Roche, US, Cat#10236276001) for 10 min. The cells that did not migrate were removed with a cotton swab, and the membrane was imaged. Cells from five random fields of view were counted.

### Matrigel tube formation study

Briefly, 200-µl aliquots of Matrigel (BD Sciences, Cat#354234) solution were poured into 48-well plates, and then the plates were incubated at 37°C for 30 min in a 5% CO_2_ incubator. HRMECs and HUVECs (5×10^4^ per well) were treated with 10^−4^ mg/ml and 10^−6^ mg/ml of ES, M-ES, or PEG-M-ES and 20 ng/ml of VEGF for 48 h. The cells were seeded on the Matrigel and cultured for another 12 h. The experimental conditions described here is a 2-D assay lasting for 12 h, which captures endothelial migration and morphogenesis. The networks in the Matrigel from five randomly chosen fields were counted and photographed under a microscope. The experiments were performed in triplicate and repeated three times.

### Retinal pharmacokinetic profile

In total, 105 New Zealand rabbits were divided into three groups: PBS, 5 mg/ml of M-ES, and 5 mg/ml of PEG-M-ES. After the induction of deep anesthesia, the pupils of the rabbits were dilated, and an ocular anesthetic was applied. Single intravitreal injections of 50 µl of the indicated agents were performed in the right eyes, and the left eyes were used as the normal controls. Retinal concentrations were measured at 0 h, 12 h, 24 h, 48 h, 6 d, 12 d, and 45 d following the intravitreal injection. The Human Endostatin Quantikine Enzyme-Linked Immunosorbent Assay (ELISA) kit (R & D, Cat# DNST0, Minneapolis, MN, US) was used to detect the retinal concentration of endostatin according to the manufacturer's instructions. Pharmacokinetic parameters were calculated concurrently.

### Induction of oxygen-induced retinopathy (OIR) in the mouse model and assessment of angiography

Postnatal day 7 (P7) C57BL/6 pups were exposed to hyperoxia (75% oxygen) for 5 d. On P12, the animals were returned to normoxia (room air) to induce retinal neovascularization for 5 d. At P12, the OIR mice were injected intravitreally with 1.5 µl of ES, M-ES, or PEG-M-ES at a 5 µg/µl concentration, and the controls were injected with ranibizumab (LUCENTIS, Novartis, USA) at a 10 µg/µl concentration. At P18, the mice were perfused through the left ventricle with 0.5 ml of PBS containing 50 mg of 2×10^6^ molecular weight fluorescein-dextran-FITC (Sigma, St. Louis, MO, US). After fixation, the retinas were flat-mounted. The retinas were viewed by fluorescence microscopy (Zeiss Axiophot, Thornwood, NY, US) and photographed. The non-perfused areas were analyzed with ImageJ software. The ratio of the non-perfusion area to the entire retinal area was determined.

### Induction of mouse choroidal neovascularization (CNV) models and assessment of angiography

CNV was induced by laser photocoagulation (Coherent 130SL, Coherent, Santa Clara, CA, USA) in adult female C57BL/6J mice via the following settings: 532 nm, 150 mW, 100 ms, and 100 mm. Three or four lesions were created in one eye, and the other eye was used as a control. After establishing the CNV model, 5 mg/ml of ES, M-ES, or PEG-M-ES and 5 mg/ml Ranibizumab was intravitreally injected in a 2-µl volume into each eye. On day 14 after laser photocoagulation, fluorescein angiography (FA) was performed using a digital imaging system (Phoenix Micron IV Retinal Imaging Microscope, Pleasanton, CA, US) according to the manufacturer's instructions. Thirty microliters of 5% fluorescein was administered by intraperitoneal injection after the mice were anaesthetized. Late-phase (5 minutes after injection) fundus angiograms were analyzed. The fluorescein leakage area for each lesion was measured using ImageJ software.

### Statistical evaluation

Data analysis was performed using the following statistical software programs: Prism 5 (GraphPad Software Inc., San Diego, CA, US) and SPSS (SPSS, version 16.0; SPSS Science, Chicago, IL). All data are presented as the mean ±SEM, and the normality of the distribution was assessed. Individual group means were compared using Student's unpaired t-test, and data sets were examined by one-way analysis of variance (ANOVA) followed by a post-hoc Dunnett's t-test. For group comparisons, mixed linear models were used. A p-value <0.05 was considered indicative of a statistically significant difference.

## Results

### Mutant endostatin (M-ES) and PEGylated-mutant endostatin (PEG-M-ES) preparation synthesis

**Figure 1:**
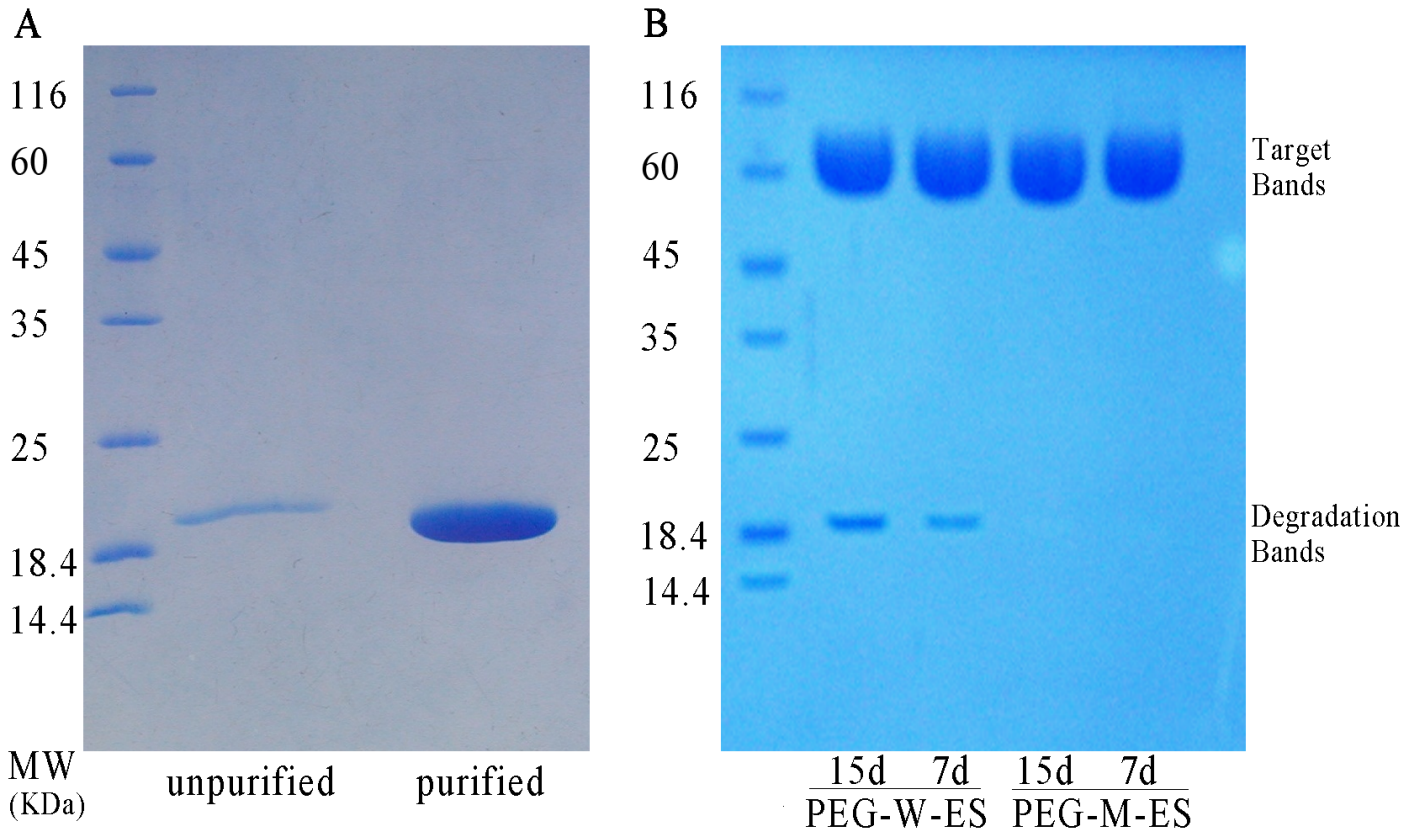
SDS-PAGE for M-ES purification and PEG-M-ES stability. Panel A Purified M-ES. Lane 1: Marker, followed by 116, 66.2, 45, 35, 25, 18.4, and 14.4 KD; Lane 2: unpurified M-ES; Lane 3: purified M-ES. Panel B The degradation rate of PEG-M-ES is significantly lower than the degradation rate of PEG-W-ES.

The recombinant vector pET-30a (+)-M-ES was confirmed by NdeI and EcoRI restriction enzyme digestion. After purification, renaturation, and repurification, the purity of endostatin achieved 95% (Figure 1, Panel A). The M-ES nucleotide sequence is presented in Table 1, and the M-ES protein sequence is shown in Table 2. According to the results, 20 kDa PEG was covalently bound to the recombinant M-ES and wild type endostatin (W-ES) at the N-terminus. After the cation exchange column was used to separate and purify PEG-W-ES and PEG-M-ES, the collected proteins were detected by SDS-PAGE (Figure 1, Panel B, target bands).

**Table 2:** M-ES amino acid sequence.

DSDRDFQPVLHLVALNSPLSGGMRGIRGADFQCFQQARAVGLAGTFRAFLSSRLQDLYSIVRRADRAAVPIVNLKDELLFPSWEALFSGSEGPLKPGARIFSFDGKDVLRHPTWPQKSVWHGSDPNGRRLTESYCETWRTEAPSATGQASSLLGGRLLGQSAASCHHAYIVLCIENSFMTASK

### Zinc ion-binding capacity was impaired in M-ES

The zinc-binding capacity is an important aspect of the structural stability of endostatin. In the present study, the histidine residues in the N-terminus of the protein at amino acid positions 1 and 3 were mutated into aspartic acid (H1D/H3D). This mutation comprehensively reduced the zinc-binding capacity of M-ES compared with that of recombinant W-ES at 10 h after incubation. The zinc ion concentration of M-ES was 0.97±0.07 µM, and the concentration of W-ES was 0.18±0.03 µM; the difference between the two groups is statistically significant (p<0.01).

### PEG stability test for PEG-W-ES and PEG-M-ES

Figure 1 (Panel B) indicates that PEG-W-ES was degraded in a time-dependent manner. On day 7, the degradation rate in the PEG-W-ES group was 5.07±0.23%. In contrast, only 0.04±0.03% was degraded in the PEG-M-ES group, which is significantly different from the PEG-W-ES group (p<0.01). On day 15, the degradation rate was much higher in the PEG-W-ES group 8.98±0.21% than in the PEG-M-ES group 0.05±0.03% (p<0.0001). Thus, the results indicate that the N-terminal mutation of ES is more stable than the wild type endostatin. In addition, PEG-M-ES is more stable than PEG-W-ES.

### The effect of ES, M-ES, and PEG-M-ES on endothelial cell proliferation

**Figure 2:**
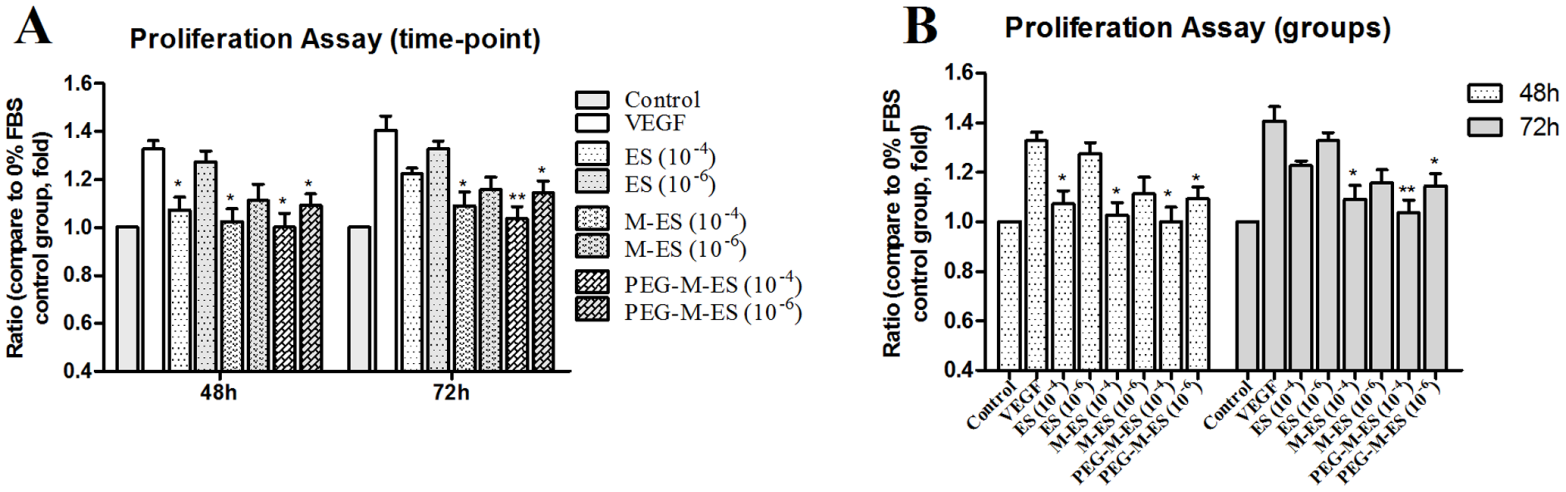
The effects of ES, M-ES, and PEG-M-ES on HRMEC proliferation in VEGF-containing culture medium. Cell proliferation was measured using the BrdU assay at 48 and 72 h. Data are presented as the mean ±SD. Each experiment was repeated at least three times. All treatment groups were compared with the group treated with VEGF_165_ (20 ng/ml). One-way analysis of variance (ANOVA) followed by a post-hoc Dunnett's t-test was used to analyze the data. *P<0.05; **P<0.01.

A proliferation study was used to evaluate the anti-angiogenic effects of ES, M-ES, and PEG-M-ES *in vitro*. As indicated in Figure 2 and Figure S1, ES inhibited the proliferation of HRMECs and HUVECs only at the concentration of 10^−4^ mg/ml, and the inhibitory effect was reduced after 72 h. M-ES inhibited HRMECs proliferation only at the concentration of 10^−4^ mg/ml, and PEG-M-ES effectively inhibited HRMECs and HUVEC proliferation at various time points throughout the study. As we previously reported, <0.1 mg/ml PEG did not affect HUVEC proliferation [19].

### The effect of ES, M-ES, and PEG-M-ES on endothelial cell migration

**Figure 3:**
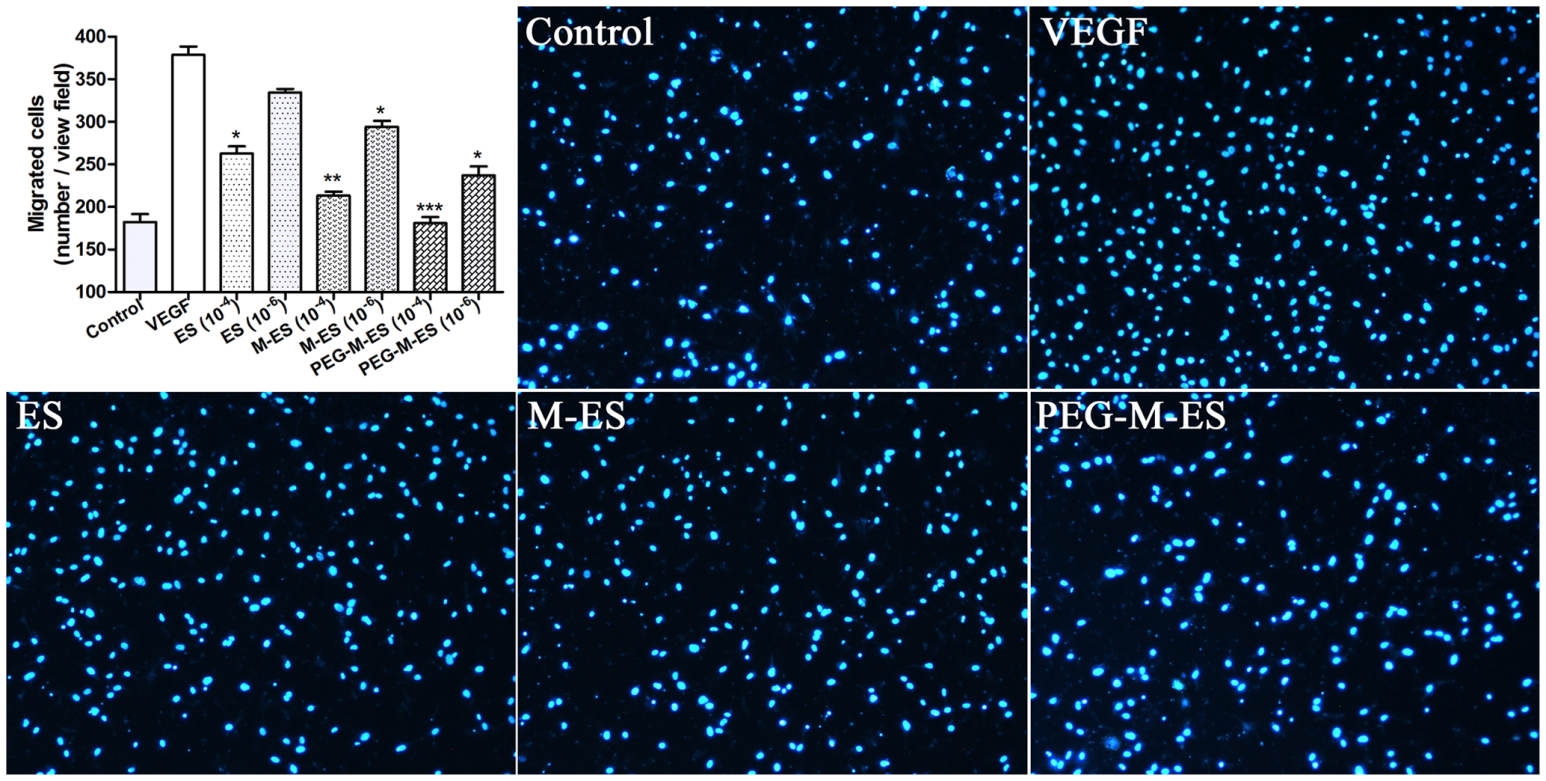
The effects of ES, M-ES, and PEG-M-ES on the migration of HRMEC s. The migratory activities of the control, the VEGF-treated group (20 ng/ml), the ES group, the M-ES group, and the PEG-M-ES group are presented. The upper left panel shows the statistical analysis results, and the other figures are representative images (10^−6 ^mg/ml agents) of various treatment groups. The cell nucleus was stained with DAPI, which is indicated in blue. Each experiment was repeated at least three times. Data are presented as the mean ±SD. One-way ANOVA followed by a post-hoc Dunnett's t-test was used to analyze the data. *P<0.05; **P<0.01; ***P<0.0001.

Migration ability was assessed with a Transwell migration assay. As shown in Figure 3 and Figure S2, the number of cells that crossed the membrane in the M-ES- and PEG-M-ES-treated HRMECs and HUVEC groups were significantly reduced compared with the number in the ES group for both the 10^−4^ and 10^−6^ mg/ml groups. The lower concentration of ES did not inhibit the migration of HUVECs.

### The effect of ES, M-ES, and PEG-M-ES on tube formation

**Figure 4:**
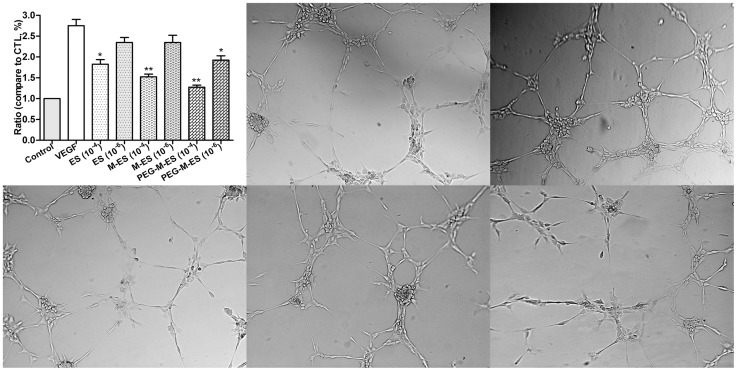
The effect of ES, M-ES, and PEG-M-ES on HRMEC tube formation. A Matrigel assay was used to evaluate the angiogenic effect of ES, M-ES, and PEG-M-ES on HRMECs. The total length of each tube in all treatment groups is presented. The upper left panel shows the statistical analysis results, and the other figures are representative images (10^−6^ mg/ml agents) of various treatment groups. Data are presented as the mean ±SD. Each experiment was repeated at least three times. One-way ANOVA followed by a post-hoc Dunnett's t-test was used to analyze the data. *P<0.05; **P<0.01.

The Matrigel assay is one of the most widely used methods to evaluate the reorganization of angiogenesis *in vitro*. In the present study, VEGF was used to enhance network formation. At the concentration of 10^−4^ mg/ml, ES, M-ES, and PEG-M-ES impaired the cell capacity to form a regular network. At the concentration of 10^−6^ mg/ml, only PEG-M-ES inhibited tube formation (Figure 4 and Figure S3).

### Long-lasting effects of PEG-M-ES in the rabbit retina

**Figure 5:**
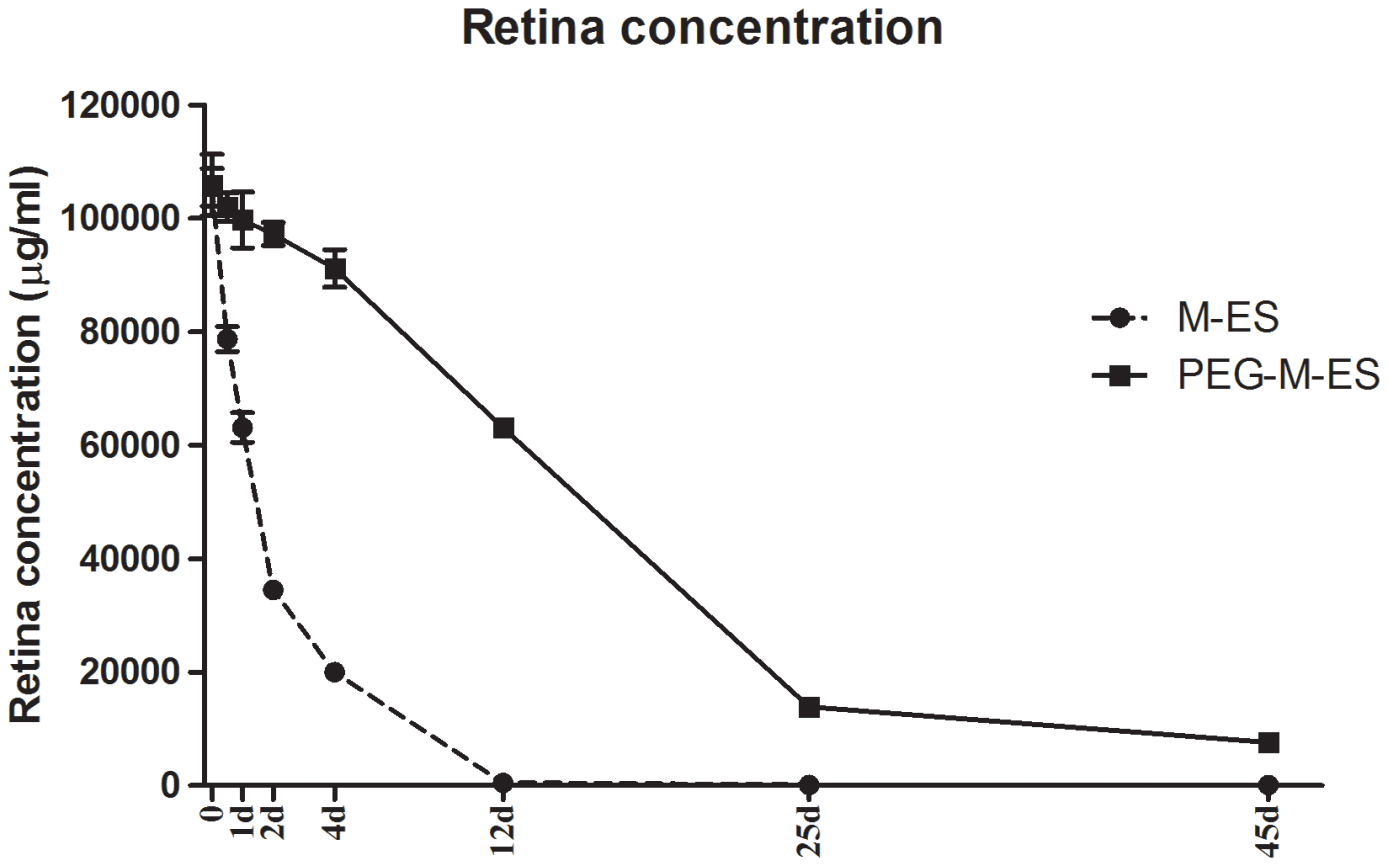
M-ES and PEG-M-ES retinal concentrations. Retinal concentrations of M-ES and PEG-M-ES following a single intravitreal injection were measured by an ELISA kit at the indicated time points. Each group contained 5 rabbits. The data are presented as the mean ±SD. The statistical method used for this assay was a mixed linear model.

To determine the *in vivo* drug metabolism rate in ocular tissue, the retinal concentrations of M-ES and PEG-M-ES after a single intravitreal injection were measured in the New Zealand rabbits using an ELISA kit at the indicated time points. As shown in Table 3, Table 4, and Figure 5, the half-lives of PEG-M-ES and M-ES were 14.67 d and 0.94 d (Figure 5), respectively, indicating that the PEG-M-ES levels were sustained in the ocular tissue compared with M-ES. We also detected other pharmacokinetic parameters, as shown in Table 4. These results indicate that PEG-M-ES exhibited a reduced degradation rate compared with M-ES *in vivo*.

**Table 3:** Retinal concentration of M-ES and PEG-M-ES in the rabbits (ng/ml).

	M-ES	PEG-M-ES
	MEAN±SD	MEAN±SD
**0 h**	105423±3320	105864±5422
**12 h**	78723±2211	102000±2522
**1 d**	63121±2628	99721±4927
**2 d**	34532±1552	97215±2024
**4 d**	20023±934	91187±3312
**12 d**	460±46	63112±1496
**25 d**	105±23	13882±724
**45 d**	85±12	7636±677

M-ES: mutant endostatin; PEG-M-ES: mutant endostatin covalently linked to PEG.

**Table 4:** Retinal pharmacokinetic parameters.

Parameters	M-ES	PEG-M-ES	p*
**T_1/2_ (d)**	0.94±0.08	14.67±1.02	<0.0001
**Ke (1/d)**	0.737±0.06	0.047±0.01	<0.0001
**V1/F (L/kg)**	0.009±0.00	0.015±0.00	<0.01
**CL/F (L/d/kg)**	0.007±0.00	0.001±0.00	<0.01
**AUC_(0-t)_ (µg/L** * **d)**	145446.445±115.2	1362757.32±521.25	0.286
**AUC_(0-∞)_ (µg/L** * **d)**	145446.449±115.3	1387366.32±533.72	0.264
**Ka (1/d)**	0.738±0.05	0.052±0.00	<0.0001
**t_1/2_Ka (d)**	0.939±0.03	13.412±0.42	<0.0001
**Tlag (d)**	0	39.019±2.12	<0.0001

M-ES: mutant endostatin; PEG-M-ES: mutant endostatin covalently linked to PEG.

*Statistical method: Student's t-test.

### The effect of ES, M-ES, and PEG-M-ES on the OIR model

**Figure 6:**
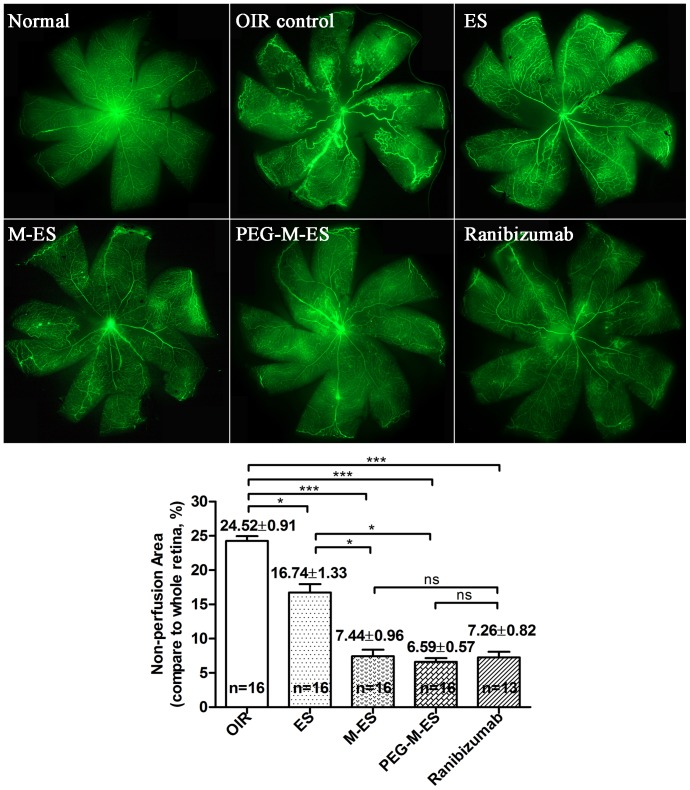
The effects of ES, M-ES, and PEG-M-ES on the OIR animal model. P7 mice were exposed to hyperoxia for 5 d. At P12, the pups were intravitreally injected with 1.5 µl of 5 mg/ml ES, M-ES, PEG-M-ES, or 1.5 µl of 10 mg/ml ranibizumab. At P18, the pups were euthanized to detect the retinal non-vascularization area. The upper left panel presents the statistical analysis results, and the other figures contain representative images of the retina FFA in various treatment groups. The data are presented as the mean ±SD. All of the measured areas were outlined. One-way ANOVA followed by a post-hoc Dunnett's t-test was used to analyze the data. *p<0.05; ***p<0.0001; ns: no significant difference.

To determine whether ES, M-ES, and PEG-M-ES had an effect on the OIR mouse model, the agents were injected intravitreally into the right eyes of the retinopathy mice at P12 (immediately after the animals were returned from hyperoxia to normoxia) and age-matched normal mice. As shown in Figure 6, ES reduced the area of neovascularization to 16.74±1.33%, which differed significantly compared with the untreated control results of 24.52±0.91% (p<0.05) (Figure 6, Figure S4). In the M-ES and PEG-M-ES treatment groups, the neovascularization area was substantially reduced to 7.44±0.96% and 6.59±0.57%, respectively, which is significantly different compared with the untreated controls (p<0.0001, for both) and the ES-treated group (p<0.05). For ranibizumab treatment group, the neovascularization area was reduced to 7.26±0.82%. There is no significant difference between M-ES, PEG-M-ES, and ranibizumab treatment groups. Thus, these experiments demonstrated that M-ES and PEG-M-ES protect the postnatal mouse retina from hyperoxia-induced neovascularization more efficiently than ES.

### Mutant endostatin protects against laser-induced CNV lesions

**Figure 7:**
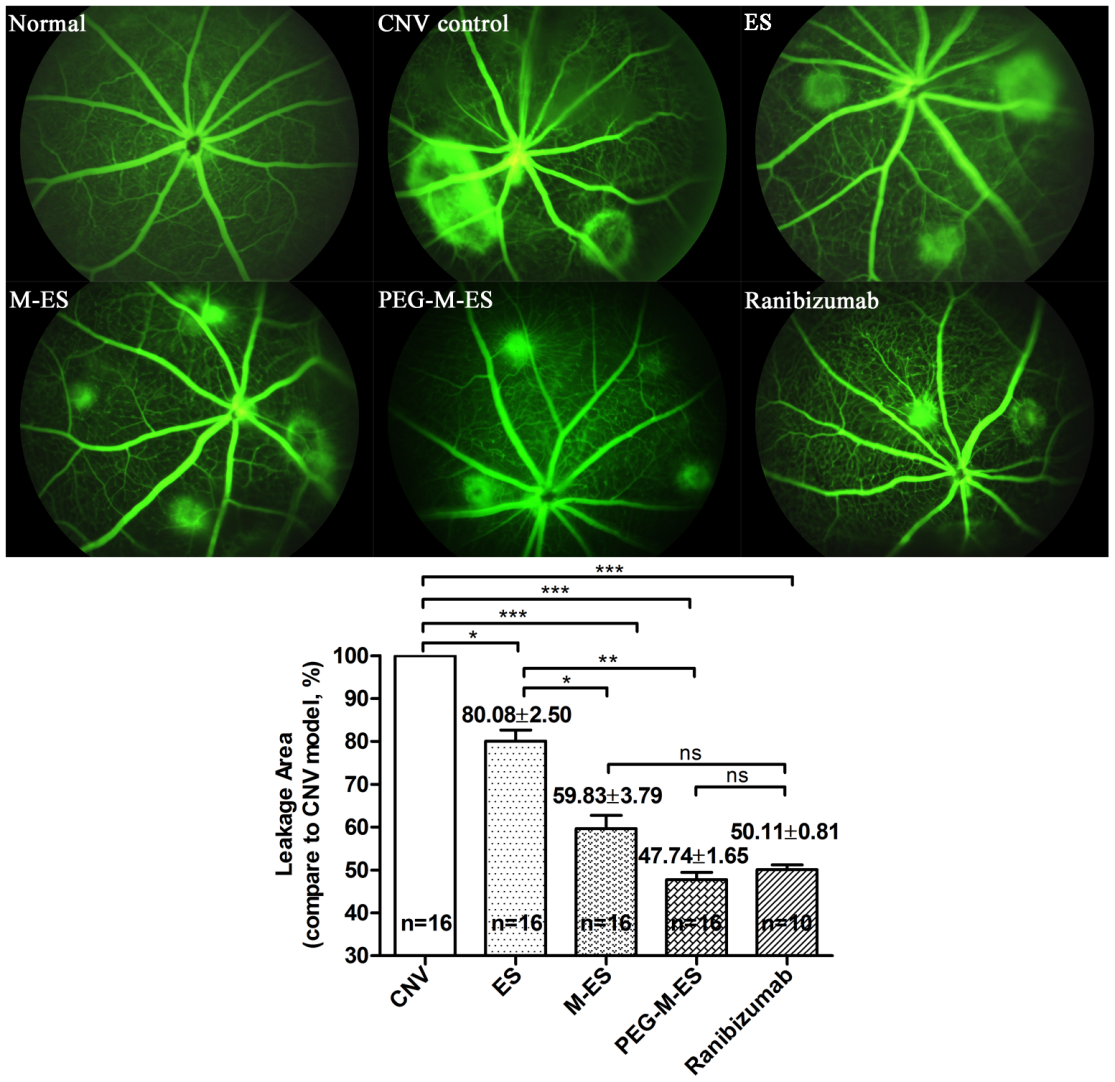
The effects of ES, M-ES, and PEG-M-ES on the CNV animal model. Angiographic analysis of CNV leakage 14 d after laser photocoagulation in the various treatment groups and the control group. The upper left panel shows the statistical analysis results, and the other figures present representative images of the retina FFA in various treatment groups. The data are presented as the mean ±SD. One-way ANOVA followed by a post-hoc Dunnett's t-test was used to analyze the data. *p<0.05; ***p<0.0001; ns: no significant difference.

CNV leakage in mice was assessed by fluorescein angiography (FA) (Figure S5). The right eyes of the mice were intravitreally injected with 2.0 µl of 5 mg/ml ES, M-ES, PEG-M-ES, or 2.0 µl of 10 mg/ml ranibizumab immediately after the induction of CNV. On day 14 after CNV was induced, hyperfluorescence leakage was observed at the lesion sites in the mice. As shown in Figure 7, ES reduced the leakage area to 80.08±2.50%, which differed significantly from the untreated control (p<0.05). In the M-ES and PEG-M-ES treatment groups, the leakage area was substantially reduced to 59.83±3.79% and 47.74±1.65%, respectively, which differed significantly from the untreated controls (p<0.0001 for both) and the ES-treated group (p<0.05 and p<0.01, respectively). For ranibizumab treatment group, the leakage area was reduced to 50.11±0.81%. There is no significant difference between M-ES, PEG-M-ES, and ranibizumab treatment groups. Thus, these experiments demonstrated that M-ES and PEG-M-ES decreased the leakage of the CNV site more efficiently than did ES.

## Discussion

Retinal neovascularization is described as the development of abnormal, tiny, leaky blood vessels within the retina [20]. Complications derived from neovascularization, such as hemorrhage and retinal detachment, contribute to severe and irreversible visual loss in most cases [5], [21]. The most common causes of retinal neovascularization include ROP, AMD, high myopia macular degeneration, and diabetic retinopathy [22].

Current knowledge indicates that neovascularization results from complex interactions between factors that either stimulate or inhibit endothelial cell differentiation, proliferation, migration, and maturation [20]. Of these, VEGF has been identified as the most important factor. [23]. The development of pharmacological anti-VEGF agents, such as ranibizumab and aflibercept, is based on the understanding of the processes involved in neovascularization and has revolutionized the treatment of retinal angiogenic diseases [24]. Nevertheless, exogenous proteins are currently used clinically as anti-VEGF medications, and evidence suggests that these agents have a systemic effect [25] and on pigment epithelial tears [26]. Additionally, the inhibition of VEGF signaling in the eye may have unexpected detrimental effects on choroidal vasculature and photoreceptor function. Therefore, the development of alternative strategies may be necessary [27].

Endostatin is an endogenous 20-kD proteolytic fragment of type XVIII collagen [7]. Recent studies have demonstrated that endostatin is one of the most active and safe natural inhibitors of tumor angiogenesis [28]. The mechanisms that underlie endostatin's inhibitory effects are complicated and include the following: the induction of endothelial cell apoptosis by inhibiting cyclin D1; endostatin binding to cell receptors (including nucleolin, integrins, laminin, and matrix metalloproteinase 2), which mediate anti-angiogenic activities and trigger a series of intracellular signaling cascades; and the regulation of angiogenic and anti-angiogenic factors [10]. Consequently, the many functions of endostatin that are related to the proliferation, motility, maturation, and apoptosis of endothelial cells, including intracellular signaling, are precisely modulated and ultimately inhibit angiogenesis and subsequent diseases [29].

Previously, we demonstrated that native endostatin exhibited inhibitory effects on retinal angiogenesis [16]. A reduction in the dosing frequency is beneficial for patients receiving intravitreal injections via the reduction of complications associated with frequent intravitreal injections, such as endophthalmitis [30]. Thus, our major research aims involve endostatin stabilization and the design of appropriate protein carriers to reduce dosing frequency. In the present study, we synthetized a biochemically stable N-terminal H1D/H3D M-ES and a 20-kDa PEG that is covalently bound to M-ES (PEGylated-M-ES, PEG-M-ES) with the aim of obtaining a long-lasting effect of endostatin in the retina.

The zinc-binding capacity is critical not only for the structural stability but also for the biological functions of endostatin [31]. Endostatin contains four zinc-binding residues (i.e., H1, H3, H11, and D76). Of these residues, H1, H3, and H11 are located in the N-terminal loops, and D76 is located in the loop between strands E and F [10]. Our M-ES contains a mutation in the first and third amino acids at the N-terminus of endostatin (H1D and H3D). The zinc-binding capacity of the mutant endostatin was impaired without impacting stability based on zinc-binding experiments (W-ES vs. M-ES: 0.18±0.03 µM compare to 0.97±0.07 µM). Although Beohm et al. reported that removing zinc ions from endostatin induced conformational changes such that endostatin became sensitive to degradation by a protease [32], our degradation assays suggested that the mutant endostatin is stable (Figure 1, panel B, degradation rate PEG-W-ES vs. PEG-M-ES: 5.07±0.23% compare to 0.04±0.03% (day 7); 8.98±0.21% compare to 0.05±0.03% (day 14)). This result may be explained by an alternative zinc-binding pattern that contributes to anti-angiogenesis activity, although the mutant endostatin lacks the first two of four zinc ligands. Additionally, the H1D and H3D mutations are beneficial for enhancing the binding of PEG or other modification materials.

Our *in vitro* and *in vivo* results suggest that M-ES had enhanced anti-angiogenic effects compared with native endostatin. To prolong the retention time within the eyes and to reduce the intravitreal injection frequency, M-ES was modified with PEG. PEGylation, first described by Abuchowski, is the act of covalently coupling a PEG structure to a larger molecule [33]. Currently, PEG is one of the most widely used polymers for modifying protein therapeutics; it has many applications ranging from industrial manufacturing to medicine [34] (e.g., PEGylated interferon alfa-2a/-2b and pegaptanib, the first FDA-approved, intravitreally injected, anti-angiogenic medication [14]). The modification of proteins with PEG is a well-established technique that has several benefits, including reduced antigenicity, immunogenicity, and toxicity; an increased circulating half-life; and enhanced bioavailability, solubility, and thermal and mechanical stability [35]. In a previously published article, Bakri SJ et al. reported that the vitreous half-life of 0.5 mg intravitreal ranibizumab is 2.88 d and that the half-life of 1.25 mg intravitreal bevacizumab is 4.32 d in rabbits [36]. In the present study, the half-life of 0.25 mg intravitreal PEG-M-ES was approximately 15 d, and the half-life of M-ES was 1 d.

In the present study, we developed H1D and H3D mutant M-ES and PEG-M-ES for the first time and evaluated their anti-angiogenic activity *in vitro* and *in vivo*. We confirmed that M-ES and PEG-M-ES significantly inhibit the proliferation, migration, and tube formation of HRMECs and HUVECs compared with native endostatin under VEGF stimulation *in vitro*. *In vivo*, we used OIR and CNV animal models to evaluate the anti-angiogenic effects of M-ES and PEG-M-ES. The OIR model closely resembles ROP, and the laser-induced CNV model resembles wet-AMD. In our *in vivo* study, M-ES and PEG-M-ES significantly inhibited neovascularization in the OIR and CNV models, and the results are comparable with ranibizumab treatment group. Considering the long-lasting effects of the PEGylated mutant endostatin, we expect that the PEG-M-ES delivery system will be a promising approach for the long-term treatment of retinal neovascularization diseases.

## Supporting Information

Figure S1 **The effects of ES, M-ES, and PEG-M-ES on HUVEC proliferation in VEGF-containing culture medium.** Cell proliferation was measured using the BrdU assay at 48 and 72 h. Data are presented as the mean ±SD. Each experiment was repeated at least three times. All treatment groups were compared with the group treated with VEGF_165_ (20 ng/ml). One-way analysis of variance (ANOVA) followed by a post-hoc Dunnett's t-test was used to analyze the data. *P<0.05; **P<0.01.
(TIF)

Figure S2 **The effects of ES, M-ES, and PEG-M-ES on HUVEC proliferation in VEGF-containing culture medium.** Cell proliferation was measured using the BrdU assay at 48 and 72 h. Data are presented as the mean ±SD. Each experiment was repeated at least three times. All treatment groups were compared with the group treated with VEGF_165_ (20 ng/ml). One-way analysis of variance (ANOVA) followed by a post-hoc Dunnett's t-test was used to analyze the data. *P<0.05; **P<0.01.
(TIF)

Figure S3 **The effect of ES, M-ES, and PEG-M-ES on HUVEC tube formation.** A Matrigel assay was used to evaluate the angiogenic effect of ES, M-ES, and PEG-M-ES on HUVECs. The total length of each tube in all treatment groups is presented. The upper left panel shows the statistical analysis results, and the other figures are representative images (10^−6^ mg/ml agents) of various treatment groups. Data are presented as the mean ±SD. Each experiment was repeated at least three times. The DMEM+0% FBS control was set to 1. One-way ANOVA followed by a post-hoc Dunnett's t-test was used to analyze the data. *P<0.05; **P<0.01.
(TIF)

Figure S4 **The method for measuring non-perfusion area in OIR model.** The non-perfusion areas were outlined by yellow circle.
(JPG)

Figure S5 **The method for measuring leakage area of CNV model.** The leakage areas were outlined by yellow circle.
(JPG)
