# Supplementary figures and images for: Anti-Angiogenic Effects of a Mutant Endostatin: A New Prospect for Treating Retinal and Choroidal Neovascularization
Source: PLoS One. 2014 Nov 7;9(11):e112448. doi: 10.1371/journal.pone.0112448 (PMC4224489; doi:10.1371/journal.pone.0112448)

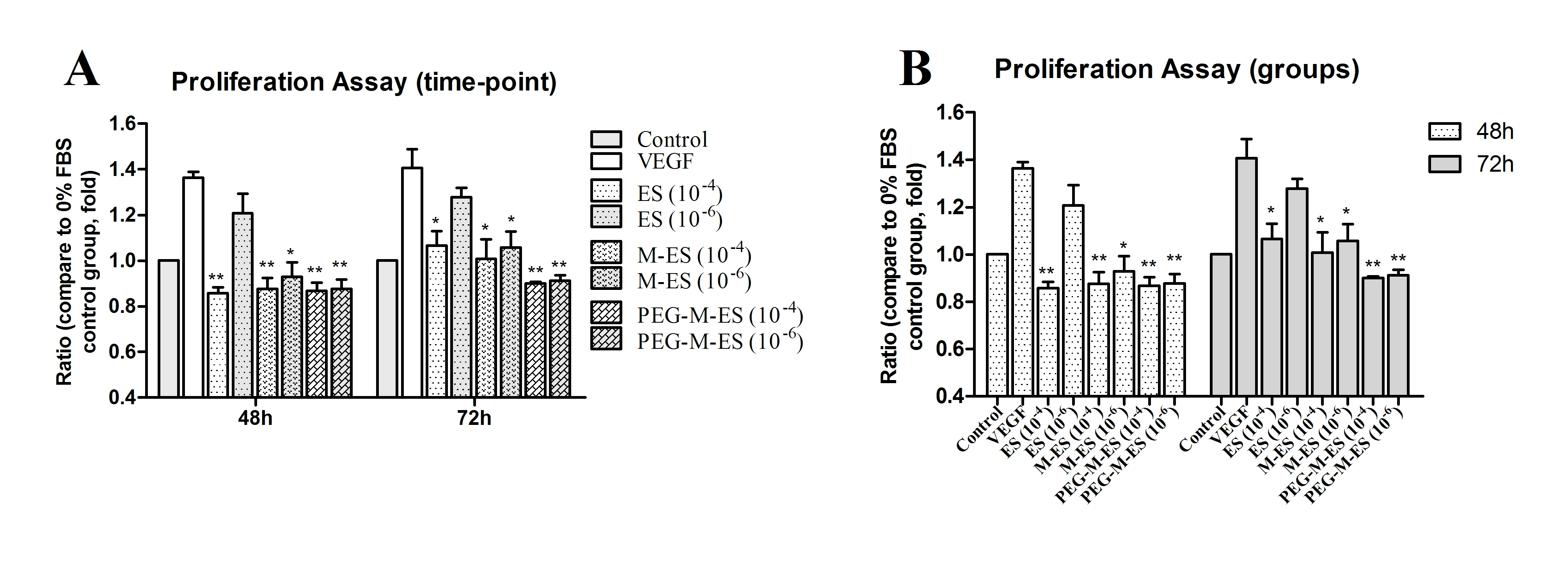

Supplement: Figure S1 — The effects of ES, M-ES, and PEG-M-ES on HUVEC proliferation in VEGF-containing culture medium. Cell proliferation was measured using the BrdU assay at 48 and 72 h. Data are presented as the mean ±SD. Each experiment was repeated at least three times. All treatment groups were compared with the group treated with VEGF165 (20 ng/ml). One-way analysis of variance (ANOVA) followed by a post-hoc Dunnett's t-test was used to analyze the data. *P<0.05; **P<0.01. (TIF) [file pone.0112448.s001.tif]

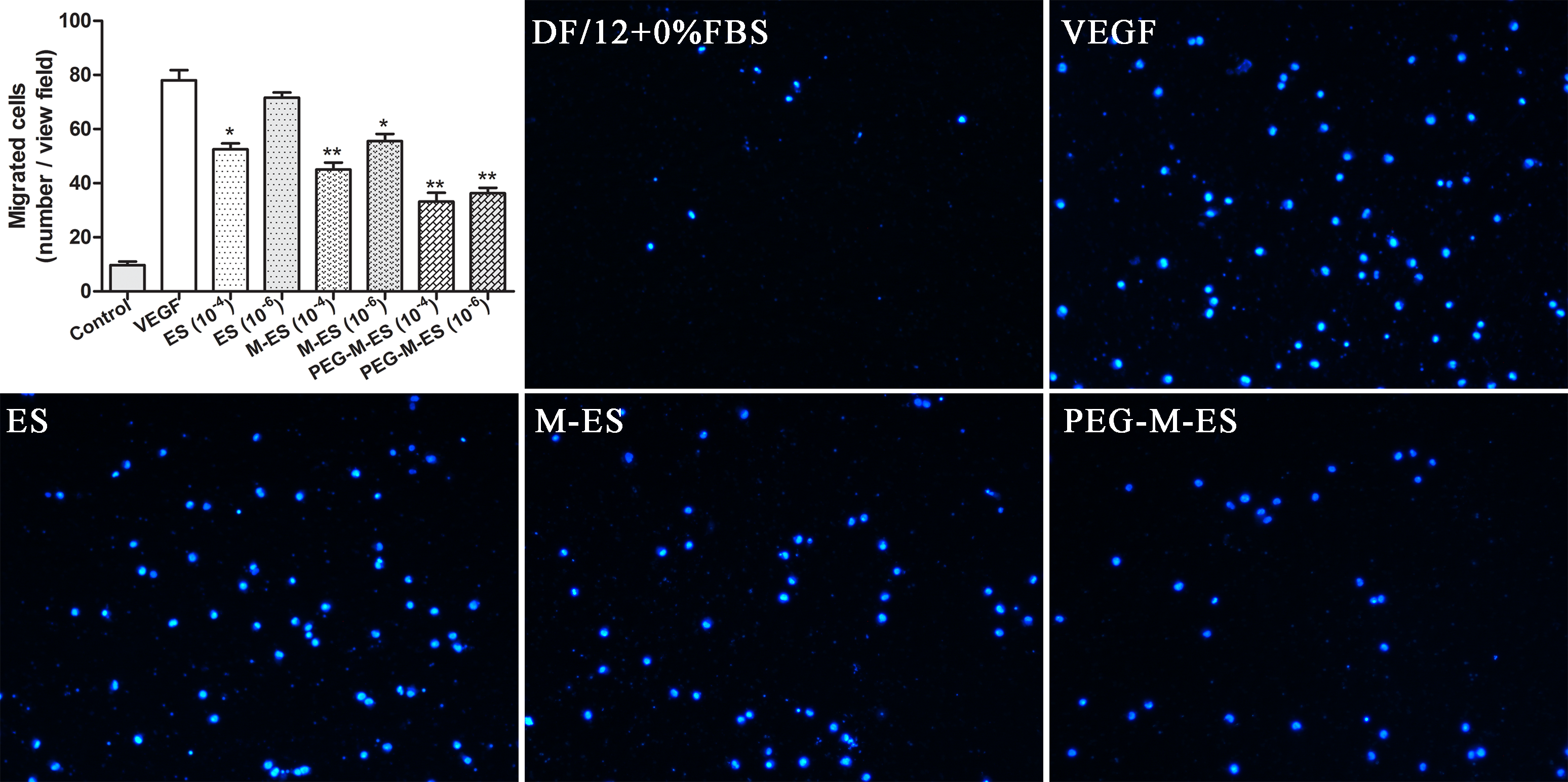

Supplement: Figure S2 — The effects of ES, M-ES, and PEG-M-ES on HUVEC proliferation in VEGF-containing culture medium. Cell proliferation was measured using the BrdU assay at 48 and 72 h. Data are presented as the mean ±SD. Each experiment was repeated at least three times. All treatment groups were compared with the group treated with VEGF165 (20 ng/ml). One-way analysis of variance (ANOVA) followed by a post-hoc Dunnett's t-test was used to analyze the data. *P<0.05; **P<0.01. (TIF) [file pone.0112448.s002.tif]

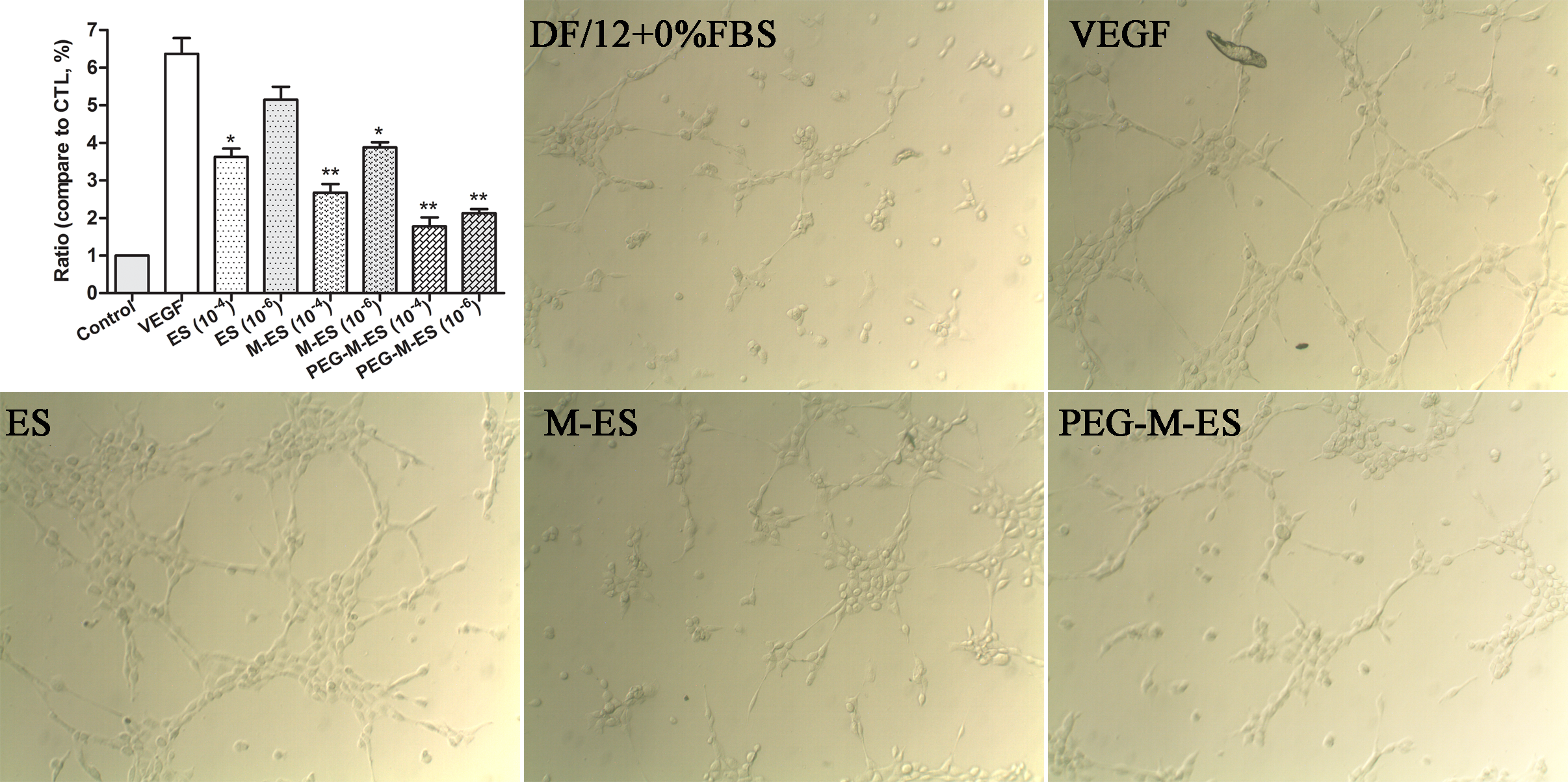

Supplement: Figure S3 — The effect of ES, M-ES, and PEG-M-ES on HUVEC tube formation. A Matrigel assay was used to evaluate the angiogenic effect of ES, M-ES, and PEG-M-ES on HUVECs. The total length of each tube in all treatment groups is presented. The upper left panel shows the statistical analysis results, and the other figures are representative images (10−6 mg/ml agents) of various treatment groups. Data are presented as the mean ±SD. Each experiment was repeated at least three times. The DMEM+0% FBS control was set to 1. One-way ANOVA followed by a post-hoc Dunnett's t-test was used to analyze the data. *P<0.05; **P<0.01. (TIF) [file pone.0112448.s003.tif]

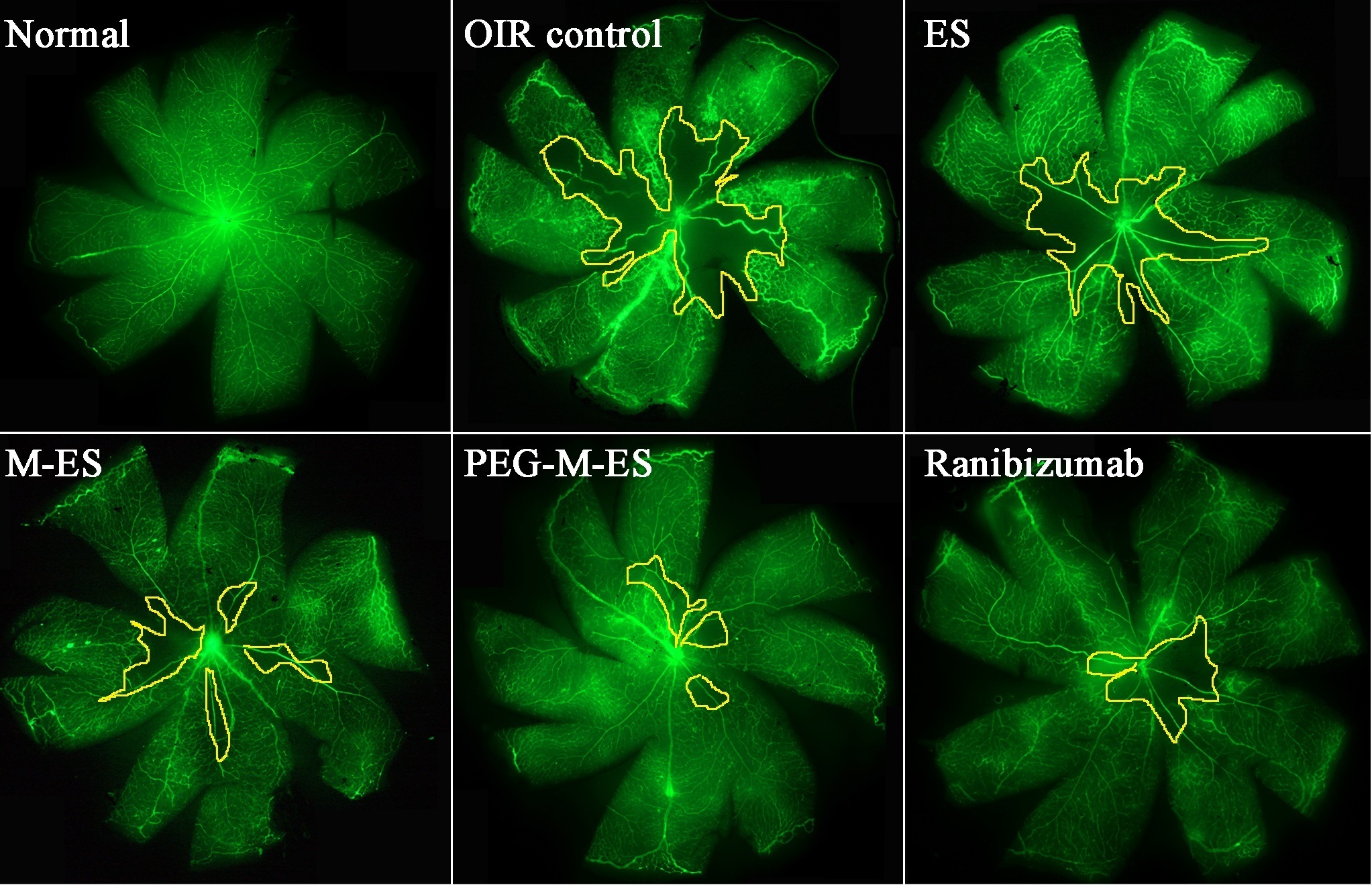

Supplement: Figure S4 — The method for measuring non-perfusion area in OIR model. The non-perfusion areas were outlined by yellow circle. (JPG) [file pone.0112448.s004.jpg]

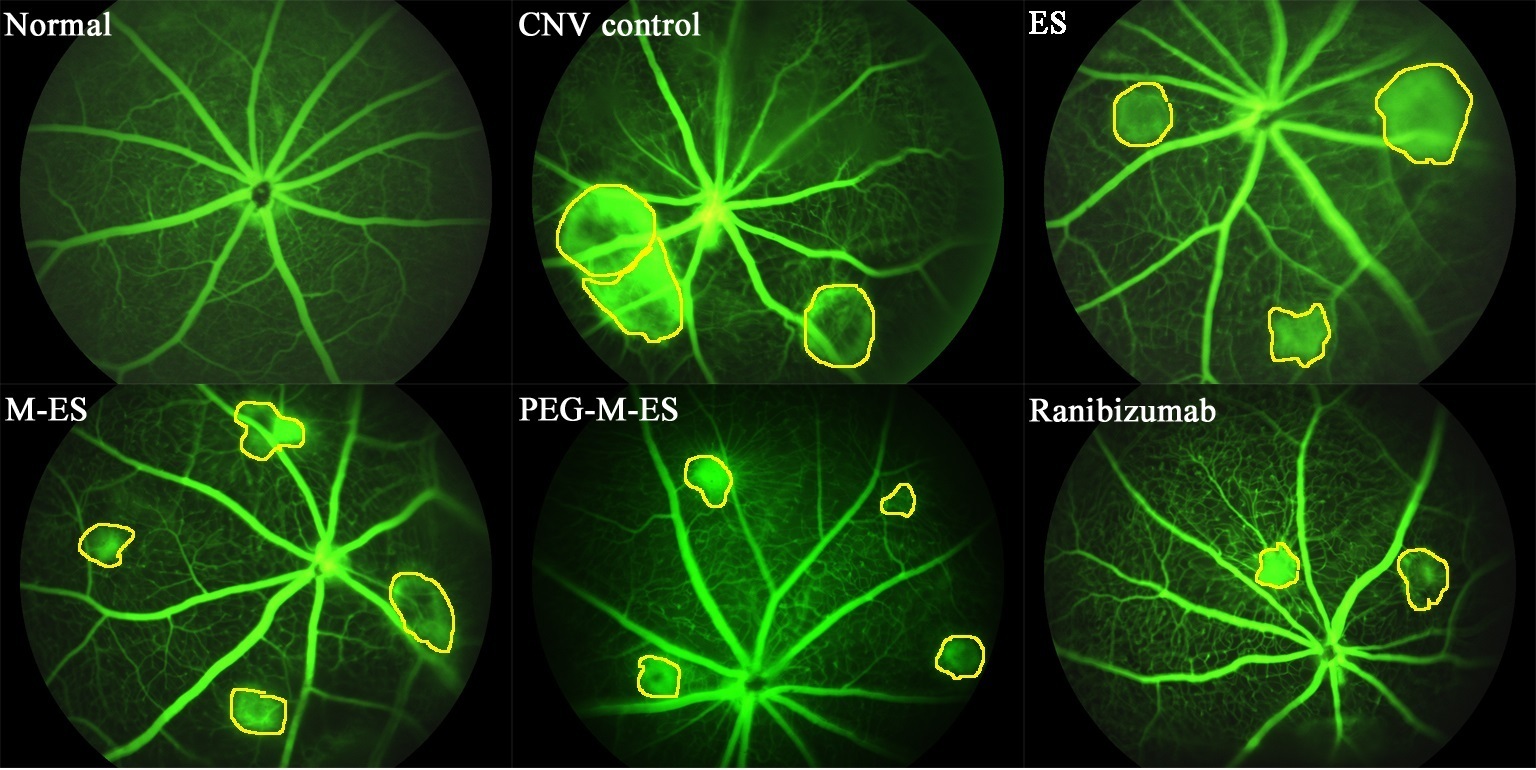

Supplement: Figure S5 — The method for measuring leakage area of CNV model. The leakage areas were outlined by yellow circle. (JPG) [file pone.0112448.s005.jpg]
